# A Random Differential Equation Approach for Modeling the Growth of Microalgae in Photobioreactors

**DOI:** 10.1007/s11538-026-01609-3

**Published:** 2026-02-25

**Authors:** C. Andreu-Vilarroig, J.-C. Cortés, A. Navarro-Quiles, Š. Papáček, C.-L. Pérez

**Affiliations:** 1 Instituto Universitario de Matemática Multidisciplinar, Universidad Politécnica de Valencia, Camino de Vera s/n, Valencia, 46022 Spain; 2 Institute of Information Theory and Automation, Czech Academy of Sciences, Prague, Czech Republic

**Keywords:** Biological system modelling, Microalgae, Photobioreactor, Industrial control, Uncertainty Quantification, Random differential equations, Simulations

## Abstract

The three-state photosynthetic factory model is frequently employed to analyze microalgal growth in photobioreactors, wherein cells continuously transition between light and dark areas. Experimental data indicate that hydrodynamic mixing results in non-constant, randomly varying intervals between successive light-dark transitions. To address this characteristic, we reformulate the deterministic model as a random differential equation, regarding the switching period as a positive random variable. We derive closed-form expressions for the long-term mean and variance of the model, showing that the average random model differs from the quasi-steady periodic deterministic trajectory. Monte Carlo simulations are utilized to illustrate how the distribution of switching periods and the time fraction spent in darkness influence productivity. The simulations show that, if the average irradiance per cycle matches the optimum under continuous illumination, rapid flashing maintains high productivity even under highly variable periods, while slower and irregular cycles can lead to significant losses, with adjustments to dark fractions having a relatively minor effect. To the best of our knowledge, this work provides the first systematic application of a random differential equation framework to a PSF-type model for microalgal growth under intermittent light regimes, and offers quantitative guidance for the design and operation of photobioreactors under realistic, highly variable light conditions.

## Introduction and motivation

Microalgae play an important role in modern biotechnology due to their potential in the large-scale production of biofuels, animal feed, and high-value bioactive compounds for pharmaceutical and nutraceutical applications (Tredici 2010; Mata et al. 2010; Mulluye et al. 2023). Their versatility has motivated a wide range of cultivation strategies and modeling approaches across industrial, agricultural, and environmental domains. For example, mathematical models have been developed to examine microalgae’s role in agriculture, such as nutrient recovery and soil stabilization through detritus reuse (Mahmood et al. 2023), while other models have focused on controlling harmful algal growth in ecological systems using stochastic control methods (Yoshioka and Yaegashi 2019).

Supporting these diverse applications that rely on microalgae requires the ability to cultivate them in a continuous and optimized manner. Among the most promising avenues for commercial algae production are closed indoor photobioreactors (PBRs), which offer enhanced control over environmental variables such as light, temperature, and contamination. Although more expensive to construct and operate than open (usually outdoor) systems, their efficiency and reliability make them attractive for industrial applications (Tredici 2010).

In this work, we focus on modeling microalgae growth dynamics specifically within PBRs, where optimizing productivity under uncertain operating conditions is a key challenge. The main difficulty in modeling of biophysical phenomena in PBRs is to find a sufficiently accurate modeling framework for the coupling between physics and biology, i.e., how to describe simultaneously the transport phenomena (a multiphase algae-water-gas flow), radiative light transfer, and bioreaction kinetics. Given the fact that the microalgae concentration in PBRs is usually low and small cells are well dispersed in medium, the first simplification consists in considering only one liquid phase. The second reasonable simplification concerns the microalgae cell buoyancy: In general most of microalgae strains are slightly denser than the surrounding medium and thus exhibit a small net sinking velocity (typically of the order 0.1–3 m $\text {day}^{-1}$ in natural phytoplankton communities), rather than being strictly neutrally buoyant. However, in well-mixed PBRs this weak negative buoyancy is usually overwhelmed by the flow regime within PBRs, more precisely by the turbulent mixing, so that unicellular microalgae can be treated to a good approximation as passive, nearly neutrally buoyant tracers. Another highly adopted simplification consists of light averaging and treating PBRs as well mixed system with lumped parameters, where the reaction kinetics is represented as the steady state relation between light intensity and the rate of photosynthesis, the so-called *P–I* curve, see e.g., Richmond and Hu (2003),[8]. This oversimplification in fact cancels the importance of dynamic time dependent phenomena, such as intermittent light regime, and shall be clarified step by step along this paper.

**Fig. 1:**
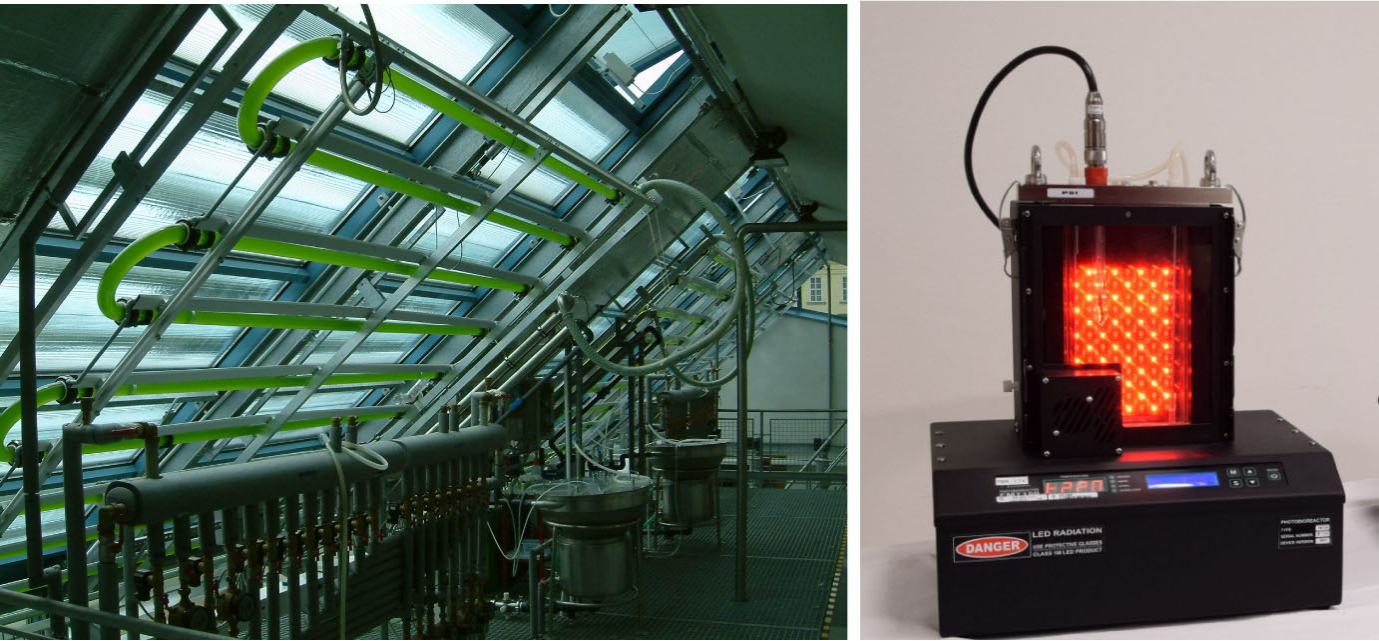
(Left) Tubular photobioreactor with Fresnel lenses for indoor cultivation of microalgae under supra-high irradiance; designed and built at the University of South Bohemia, Czech Republic (Masojıdek et al. 2003). (Right) Laboratory photobioreactor FMT 150, made by Photon Systems Instruments, Czech Republic (www.psi.cz) enabling a dynamic regulation of operating conditions (light regime, temperature, and gas composition) according to a user defined protocol (Rehák et al. 2008)

Predominantly, there are two modes of PBRs operation: (i) Batch and (ii) Continuous, see e.g., [8]. For simplicity, we further consider the continuous mode only, i.e., the biomass concentration is kept constant by removing the cell culture and feeding the fresh medium. Although the complete description of the biotechnological details of microbial cultivation is out of the scope of this paper, we further introduce the notion of *productivity* and point out the distinction between cell number density and biomass concentration. We denote by *N*(*t*) the cell (number) density of the microalgae population [typically $10^6$–$10^8$ cells $\text {mL}^{-1}$ in lab-scale cultures]. Then, by $c_x(t)$ we denote the algal biomass concentration expressed as dry weight [typically 0.1–5 g DW $\text {L}^{-1}$ in dense photobioreactor cultures].^1^ The (overall) productivity $P_o$ of a general bioreactor operated in continuous mode, which is proportional to bioreactor volume *V*, biomass concentration $c_x$ and specific growth rate $\mu$ ($\mu :=\frac{ \mathrm dc_x }{\mathrm dt}\frac{1}{c_x}$), i.e., $P_o \sim \mu ~c_x ~V$. In order to maximize the productivity, there is a strong pressure to operate PBRs with (ultra) high cell density cultures (Richmond and Hu 2003). Let us underline, unlike to conventional heterotrophic bioreactors, where a substrate and nutrients can be proportioned almost without restrictions in the whole culture volume, the light intensity (mostly representing the limiting substrate for an algal culture), which is continuously entering through the reactor wall, is exponentially decreasing in direction of light gradient according to the Beer-Lambert law, forming the zones with both supra and sub optimal irradiance (Richmond and Hu 2003). Therefore, microalgae cells while traveling through the light and dark zones of a bioreactor, either due to the pressure provided by a pump, see Fig. 1 (left), or by the bubbling in bubble column reactors, undergo light and dark periods of variable lengths, i.e., perceive the light intermittently.

The microalgae photosynthesis under intermittent light regime, so-called light/dark cycles, has been studied since the early 1930s, see the seminal work of Emerson and Arnold (1932), where the authors used short light pulses separated by dark intervals on green microalga *Chlorella* to show that oxygen evolution requires a sequence of photochemical and slower “dark” steps rather than a single photon event. Several decades later, Kok in Kok (1953) refined this picture using carefully timed flash/dark sequences. This line of investigation led to laboratory studies of microalgal photosynthesis under controlled light/dark cycles (also called as flashing light experiments) in specialized devices with time scales varying from millisecond to second, see the works of Terry Terry (1986) and Nedbal Nedbal et al. (1996).

While the possibility to induce a precise light/dark cycles, defined by the frequency, light/dark ratio, and the incident (or average) irradiance, is characteristic of small, specialized laboratory equipment, see Fig. 1 (right), in most natural and engineered environments (such as PBRs), light is not delivered uniformly across the entire system; instead, the intensity and duration of light perceived by cells fluctuate due to the cell movement across the scalar field of light intensity. Both experimental and theoretical studies indicate that many microalgae can sustain near-optimal growth under intermittent lighting, provided the flash frequency lies in a species-specific range (Janssen 2002; Papáček et al. 2007). There is also evidence that dark phases at sufficiently high frequency can help cells recover from photoinhibition (Merchuk et al. 2000). In this context, various models have been proposed to describe and study the intracellular light response of microalgae, including the widely used three-state photosynthetic-factory (PSF) model (Eilers and Peeters 1988; Wu and Merchuk 2001). There exist similar models for algal growth under light/dark cycles, in which the photosynthetic units are partitioned into resting, active and inhibited fractions, see e.g. Rudnicki et al. (2017), where six dynamic models (including PSF model) are compared. The common feature of the mechanistic photosynthetic factory models is the concept of photosynthetic units (PSU). These PSUs can be activated from the resting state by light absorption. Then, in the presence of high irradiance, the activated PSU can be photodamaged causing the transfer to the photoinhibited PSU state. By the dissipation of the excessive energy the PSU transfers from the photoinhibited to the inactive (resting) state again. The growth associated process, the so-called Calvin–Benson cycle (Richmond and Hu 2003), proceed in low light conditions, which is modeled via the transfer from the activated PSU state to the resting state.

Although the long-term objective in this line of research is to maximize the microalgae growth rate, the specific goal of the present paper is more restricted: we investigate how random variability in the period of light/dark cycles influences the long-time behaviour of the model and the associated productivity (measured by the specific growth rate), and how this compares to a purely periodic deterministic forcing. We therefore do not formulate or solve an optimal control problem here.

Let us once again emphasize that, empirical evidence suggests that the frequency at which photosynthetic cells perceive light is inherently random, particularly in industrial settings (Chiarini and Quadrio 2021), leading to fluctuations in growth dynamics that cannot be adequately captured by a purely deterministic model. Faced with this issue, in the setting mathematical modelling of engineering problems with differential equations, there are two main approaches for the mathematical modeling taking into account uncertainty, namely, stochastic differential equations (SDEs) and random differential equations (RDEs). It is worth emphasizing that there is a growing tendency in the uncertainty quantification literature to use the terms SDE and RDE interchangeably, despite the fact that they represent fundamentally different mathematical formulations and require distinct analytical and numerical techniques, as underscored in (Smith, 2014, p. 96).

SDEs add a continuous-time noise term (typically Brownian motion) directly to the deterministic dynamical equations. SDEs are particularly suitable when randomness manifests as rapid, small-scale perturbations of the system state, as typically occurs in financial volatility models (Jeanblanc et al. 2009) or epidemiological models where transmission rates fluctuate randomly at short time scales (Lahrouz et al. 2017). An alternative and often more natural strategy, when uncertainty arises from intrinsic variability in model parameters rather than state-driven noise, is to utilize RDEs (Soong 1973; Neckel and Rupp 1624). In a RDE, selected parameters are modeled as random variables (or slowly varying random processes), while the system evolves according to an ordinary differential equation conditional on these parameters. This framework allows us to incorporate physical variability in light/dark cycles encountered in industrial settings without altering the well-established structure of the deterministic PSF model but incorporating the random nature of the corresponding model parameter resulting in a more realistic model. The RDE framework has been widely used in biological and ecological modeling when uncertainty arises through heterogeneous physiological properties or environmental parameters rather than instantaneous stochastic forcing via different techniques as generalized polynomial chaos (Reimer et al. 2022), collocation (Bertaglia et al. 2022), random variable transformation technique (Burbank et al. 2025), Liouville-based or transport-equation approaches for propagating parametric uncertainty (Bevia et al. 2023), etc.

As it shall be explained later in detail, in our case, randomness affects the effective light perception frequency characterizing the photosynthetic machinery. Since this uncertainty naturally appears as parameter variability –rather than external perturbations to the biomass or intracellular states– an RDE formulation is more consistent and interpretable than an SDE approach. This framework therefore enables us to account for micro-scale variability in photosynthetic responses while preserving the well-established mechanistic structure of the underlying deterministic PSF model.

Based on this justification, in this work, we reformulate into a RDE model the three-state PSF, considering the microalgae growth under intermittent light with randomly distributed light/dark cycle length (or period). Our orange randomized mathematical framework intuitively extends the PSF deterministic model by incorporating randomness into the system’s perceived light fluctuations, thereby yielding a more comprehensive representation of the photosynthetic response. In particular, we model the growth of microalgae in conditions similar to photobioreactors, where perceived light fluctuates rapidly due to hydrodynamic mixing. This random model allows us to study some random properties of the system and to quantify the experimental uncertainty of the photobioreactor’s productivity.

**Paper outline.** Section 2 develops the deterministic PSF model: it introduces the full system of ordinary differential equations and its square-wave irradiance reduction (§2.1), defines the productivity metric (§2.2), and derives the periodic solution (§2.3). Sect. 3 extends the framework to random flashing periods, establishes long-term behavior for the mean (§3.1) and variance (§3.2), and quantifies the impact of period variability on productivity via Monte-Carlo simulations (§3.3). Conclusions are drawn in Sect. 4.

## Deterministic three-state photosynthetic factory (PSF) model: A brief overview

For the sake of completeness, prior to integrating randomness into the model, we will undertake a comprehensive review of the deterministic model, delineating its parameters, the process of model order reduction, and the boundary conditions of the reduced model.

In Subsect. 2.1 we formulate the PSF dynamics as a deterministic system of ordinary differential equations (ODEs) describing transitions between photosynthetic states and, through a re-parametrization, obtain the fast-timescale reduction given in (7). Subsection 2.2 models flashing-light experiments with a square-wave irradiance, establishes convergence to a periodic solution, and derives the average specific growth rate under cycling from which we define the productivity relative to continuous illumination. Subsection 2.3 then supplies a closed-form recursive solution for the reduced model under periodic forcing, yielding analytic bounds and a transparent description of the periodic regime. Together, these results bridge the stiff full model to an analytically tractable periodic approximation, setting the stage for the stochastic extension that follows.

### Deterministic formulation of the PSF model

**Fig. 2:**
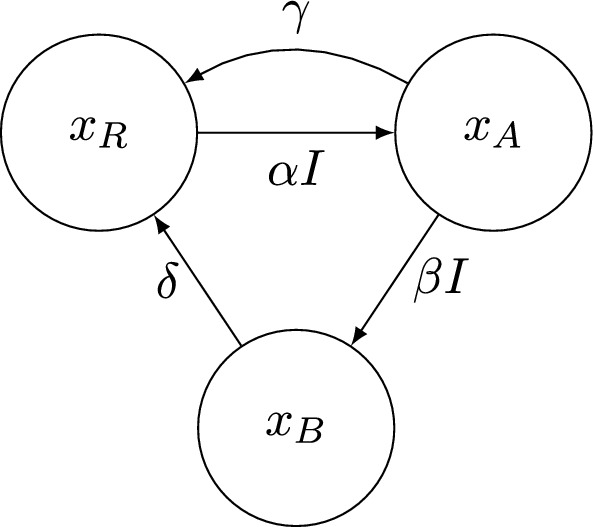
Compartmental representation of the states and transitions within the PSF model formulated in (1).

The PSF model represented in Figure 2 can be formulated via a system of ODEs that can be written in matrix form as

1 $$\begin{aligned} \begin{bmatrix} \dot{x}_{\text {R}} \\ \dot{x}_{\text {A}} \\ \dot{x}_{\text {B}} \end{bmatrix}&= \begin{bmatrix} 0 & \gamma & \delta \\ 0 & -\gamma & 0 \\ 0 & 0 & -\delta \end{bmatrix} \begin{bmatrix} x_{\text {R}} \\ x_{\text {A}} \\ x_{\text {B}} \end{bmatrix} + I(t) \begin{bmatrix} -\alpha & 0 & 0 \\ \alpha & -\beta & 0 \\ 0 & \beta & 0 \end{bmatrix} \begin{bmatrix} x_{\text {R}} \\ x_{\text {A}} \\ x_{\text {B}} \end{bmatrix}. \end{aligned}$$

In this model $x_\text {R},~x_\text {A},$ and $x_\text {B}$ are the three states of the reaction center (resting, active, and inhibited) in photosynthesis which are dimensionless and can be interpreted as the proportion of cells in that condition (Eilers and Peeters 1988).

Therefore, it holds

2 $$\begin{aligned} x_{\text {R}}+x_{\text {A}}+x_{\text {B}}=1. \end{aligned}$$

The above equation (2), in fact represents a conservation law: it says that the total population of photosynthetic units is conserved over time, i.e., they are only transition between internal states, and none enter or leave the system.

Furthermore, *I*(*t*) represents the intensity of the incident light on the system, when the term irradiance is also used (in micromoles of photons per square meter per second).^2^

The parameter values $\alpha ,~\beta ,~\gamma \text { and}~\delta$ used in this paper are provided in Wu and Merchuk (2001), where the PSF model is applied to the microalga *Porphyridium* sp. In that study, the authors determined the following parameter values for the model:

3 $$\begin{aligned} \alpha&= 1.935 \times 10^{-3} \,\mu \text {mol photons}^{-1} \text {m}^2,\,&\beta&= 5.785 \times 10^{-7} \,\mu \text {mol photons}^{-1} \text {m}^2, \nonumber \\ \gamma&= 1.460 \times 10^{-1} \,\text {s}^{-1},&\delta&= 4.796 \times 10^{-4} \,\text {s}^{-1}. \end{aligned}$$

The PSF model is completed by introducing a macroscopic quantity that links the internal photochemical dynamics to biomass accumulation. This quantity is the *specific growth rate* $\mu$, defined as $\mu := \dot{c}_x / c_x$, where $c_x$ denotes the microbial cell concentration. At this stage, $\mu$ is introduced as a model output, whose role is to connect the fast photosynthetic state dynamics to cell growth. According to Eilers and Peeters (1988); Wu and Merchuk (2001), the rate of photosynthetic production is proportional to the flux of transitions from the activated state to the resting state, i.e., to $\gamma x_A(t)$. A dimensionless proportionality constant $\kappa$ is therefore introduced as the fifth model parameter. Considering that the value of $\kappa \cdot \gamma ~$ is of order $10^{-4}$, see Wu and Merchuk (2001), and $x_A(t)$ is periodic with period *h* (see Papáček et al. (2007) for more details), we have the following relation for the specific growth rate $\mu$, according to Khalil (2001):

4 $$\begin{aligned} \mu =\frac{\kappa \gamma }{h} \int _{{0}}^{h}{x_A(t)}\textrm{d}t ~. \end{aligned}$$

Equation (4) reveals the reason why the PSF model can successfully simulate the microalgae growth in high-frequency fluctuating light conditions: the growth is described through the “fast" state ${x_A}$, hence the sensitivity to high-frequency inputs is reached.

The PSF model fulfills the following experimental observations: (i) the steady state kinetics is of *Haldane* type (also called *substrate inhibition kinetics*) [8], which encompass both the existence of an optimal value of irradiance and the photoinhibition phenomenon; (ii) the so-called *light integration* property (Terry 1986; Nedbal et al. 1996), is an inherent property of the model, i.e., as the light/dark cycle frequency is going to infinity, the value of the resulting growth rate goes to a certain limit value, which depends on the average irradiance only (Papáček et al. 2007). Moreover, practical procedures concerning the design of experiments for PSF model parameter estimation of new microalgae strains are provided in Papáček et al. (2010); Rehák et al. (2008).

Further, in our study, we will use the model parameter values provided by Merchuck in Merchuk et al. (2000) and summarized in (3) for all the numerical examples carried out in Sect. 3.3. Nevertheless, to enhance the robustness of PSF model parameter estimation, the following re-parametrization, suggested in Rehák et al. (2008), is used:

$$\begin{aligned} q_1&:= \sqrt{\frac{ \gamma \delta }{ \alpha \beta }} \,\mu \text {mol photons}~ \text {m}^{-2}~\text {s}^{-1}, ~ q_2: = \sqrt{\frac{\alpha \beta \gamma }{\delta {(\alpha +\beta )}^2}}, \nonumber \\ q_3&:= \kappa \gamma \sqrt{\frac{ \alpha \delta }{\beta \gamma }} ~ \text {s}^{-1},~ q_4:=\alpha q_1 ~ \text {s}^{-1},~ q_5 := \frac{\beta }{\alpha }, \end{aligned}$$

where $q_2$ and $q_5$ are dimensionless.

This is an important step since as indicated in Papáček et al. (2010) system (1) is stiff since the system contains multiple time scales as can be guessed from the fact that $\alpha \gg \beta$ and $\gamma \gg \delta$. This can be easily confirmed by a simple analysis. Let us write system (1) in the form $\dot{x}=Ax+I(t)B x$. From matrix *A* observe that the values of $\gamma$ and $\delta$ given in (3) correspond to a time scale of about 7 and 2038 seconds, respectively. This means one part of the system reacts on the order of seconds, while another evolves over thousands of seconds – a huge difference –. Moreover, as *A* is upper triangular, its eigenvalues are $\lambda _1^A=0$, $\lambda _2^A=-\gamma =-0.146$ and $\lambda _3^A=-0.0004796$, so the ratio of magnitudes between the fast and the slow decaying modes is $|\lambda _2^A| \, / \, |\lambda _3^A| \approx 304.4$. This eigenvalue disparity is a classic indicator of stiffness since fast and slow modes coexist. Furthermore, the presence of these vastly different eigenvalue magnitudes leads to ill-conditioning for explicit solvers. More specifically, stability conditions for explicit methods (like the forward Euler method) require time step $\Delta t$ to be small enough to resolve the fastest mode (in our case, $\Delta t < \frac{2}{|\lambda _{\text {max}}|}\approx 13.7 \text {s}$ just to ensure stability), but to resolve the slow mode (with decay on the order of $\approx 2000\text {s}$), we would need to simulate for a very long time.

Additionally, the term $I(t)B\textbf{x}$ introduces time-varying stiffness depending on the light intensity. Since *I*(*t*) scales the transition rates proportionally to $\alpha$ and $\beta$, and $\alpha \gg \beta$, this can create even faster transitions during high-light periods exacerbating the stiffness.^3^

Then, using the above-mentioned re-parametrization, the notation $u(t):=I(t)/q_1$, and equation (2), the PSF model acquires the following simplified form

5 $$\begin{aligned} \frac{1}{q_4} \begin{bmatrix} \dot{x}_A \\ \dot{x}_B \end{bmatrix}=D\begin{bmatrix} x_A \\ x_B \end{bmatrix}+u(t)M\begin{bmatrix} x_A \\ x_B \end{bmatrix} \end{aligned}$$

where

6 $$\begin{aligned} D=\left[ \begin{array}{ccc} - {q_2(1+{q_5})} & 0 \\ 0 & - \frac{q_5}{q_2(1+{q_5})} \end{array} \right] \,,\quad M=\left[ \begin{array}{ccc} - (1+q_5) & -1 \\ q_5 & 0 \\ \end{array} \right] . \end{aligned}$$

Note that given a constant and nonnegative input signal (i.e, $u \in \mathbb {R}^+$), the system of ODEs (5)-(6) is linear, and one can determine the steady-state values of states ${x_{\text {A}}}$ and ${x_{\text {B}}}$. These steady states values, denoted as $x_{\text {A}}^{\text {ss}}$ and $x_{\text {B}}^{\text {ss}}$, are

7 $$\begin{aligned} {x_{\text {A}}^{\text {ss}}(u)} =\frac{u}{ q_2(1+q_5)({u}^2+u/q_2+1)}~, \end{aligned}$$

8 $$\begin{aligned} {x_{\text {B}}^{\text {ss}}(u)} = \frac{u^2}{ u^2+u/q_2+1} ~ . \end{aligned}$$

Although the system of ODEs (5)-(6) appears simple, solving it numerically can still be challenging because of the same reasons previously exhibited for system (1). Indeed, it is enough to observe that in this case the eigenvalues of matrix *D* are $\lambda _1^D=-q_2 (1+q_5)\approx -0.3017$ and $\lambda _2^D=-\frac{q_5}{q_2 (1+q_5)}\approx -9.91\times 10^{-4}$, so differing greatly in scale, which confirms the presence of stiffness in the simplified system. In this case, the stiffness ratio (defined as the ratio of the largest to the smallest eigenvalue) is $\frac{1}{q_5}$. Specifically, the state $x_{\text {B}}$ evolves more slowly than $x_{\text {A}}$, additional model simplifications have been proposed in Papáček et al. (2010). In particular, Papáček et al. (2010) introduces two complementary order reductions, focusing on the system’s slow and fast dynamics. Suppose that $u(t)=u(t+h)$, for some period $h>0$. Let us denote the average irradiance as ${u_{\text {av}}}:=\frac{1}{h}\int _{{0}}^{h}{u(t)}\textrm{d}t$.

Then the “fast" reduction of the system of ODE (5), in terms of states $x_{\text {A}}$ and $x_{\text {B}}$, has finally the following form:

9 $$\begin{aligned} \dot{x}_{\text {A}}^{\text {F}}= -q_4 \left[ u(t)+q_2\right] {x}_A^{\text {F}} + q_4 u(t) \left[ 1 - {x_{\text {B}}^{\text {ss}}}(u_{\text {av}}) \right] , \end{aligned}$$

where the upper index “F" aims to avoid confusion with notation for the non-reduced model (5) and $x_B^{\text {ss}}$ is defined in (8). We are interested in this “fast" reduction of the system of ODE, as experimental results indicate that plant optimization is achieved when the period *h* is small (Janssen 2002; Wu and Merchuk 2001). The strategy of averaging over fast oscillations is a classical averaging or homogenization technique (Khalil 2001, Sect. 11.4–11.5). In our case, because *u*(*t*) is fast-varying (i.e., it has a short period $h>0$), but $x_{\text {B}}$ evolves slowly, we can average the fast oscillations to obtain the approximate dynamics of the slow variable. The intuition is the following: fast components like $x_{\text {A}}$ quickly reach a quasi-steady state depending on the current value of $x_{\text {B}}$ and the averaged effect of *u*(*t*). Thus, we can use a reduced model for $x_{\text {A}}$, as (9), that incorporates an averaged influence of *u*(*t*) and a frozen or slowly changing $x_{\text {B}}$. Note that in this work we are interested exclusively in the “fast” model and will now refer to $x_{A}^F$ simply as *x*.

### Normalized irradiance and growth

**Fig. 3:**
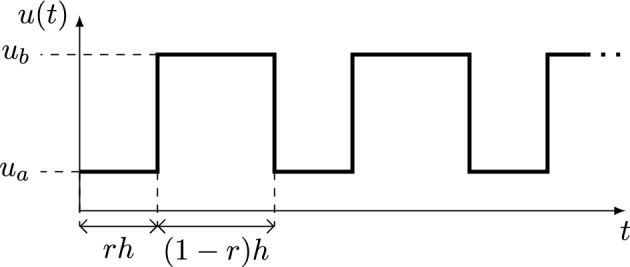
Schematic representation of the normalized effective irradiance *u*(*t*) over one light/dark cycle as defined in (10), where $u_a$ and $u_b$ denote the normalized irradiance levels in the dark and light phases and are chosen such that $u_{\text {av}}=1$.

The classical way to describe the growth of photosynthetic organisms is through the steady-state photosynthesis–irradiance (*P–-I*) curve, which assumes a spatially uniform light field. In real industrial photobioreactors, however, turbulent dispersion or bubbling continually sweeps cells between bright and dark zones, so that each cell experiences an intermittent –or flashing– irradiance pattern rather than a constant one (Papáček et al. 2018; Rehák et al. 2008). A convenient representation of this light/dark cycle is the dimensionless square-wave input

10 $$\begin{aligned} u(t)= {\left\{ \begin{array}{ll} u_a:=I_a/I_{\text {opt}}, & t\in [(i-1)h,(i-1)h+rh]=:I_1^{(i)},\\ u_b:=I_b/I_{\text {opt}}, & t\in ((i-1)h+rh,ih)=:I_2^{(i)}, \end{array}\right. } \end{aligned}$$

where $i\in \mathbb {N}$, *h* is the period, $r\in (0,1)$ is the dark fraction, $I_a$ and $I_b$ are the irradiances in darkness and light, respectively, and $I_{\text {opt}}=q_1=250~\mu \mathrm {mol~photons\,m^{-2}\,s^{-1}}$ is the optimum light level (Wu and Merchuk 2001). Note that $u(t)$ is therefore dimensionless; a schematic is shown in Figure 3. Over one cycle, the average irradiance is

$$\begin{aligned} u_{\text {av}}=\frac{1}{h}\int _{0}^{h}u(t)\,dt = u_a r + u_b (1-r), \end{aligned}$$

independent of *h*. Here, $u_a$ and $u_b$ denote the normalized effective irradiance levels during the dark and light phases, respectively, as perceived by an individual cell. In particular, $u_a=0$ corresponds to complete darkness, whereas $u_b$ is chosen such that the cycle-averaged irradiance satisfies $u_{\text {av}}=1$. In the following section, we take $u_a=0$ for simplicity. However, other values of $u_a$ could be considered to account for situations in which the dark phase is not fully dark, for instance due to partial illumination caused by reactor geometry, mixing-induced proximity to the light source, or light screening by other cells.

We now return to the definition of the specific growth rate $\mu$ introduced in Subsect. 2.1 (see expression (4)), and specialize it to the case of periodic flashing illumination. According to Eilers and Peeters (1988), $\mu$ is proportional to the flux of transitions from the activated to the resting photosynthetic state, $\gamma x_{\textrm{A}}(t)$. Once the PSF dynamics have converged after *K* cycles to a quasi-steady periodic regime under a light/dark cycle of period *h*, the cycle-averaged specific growth rate can be evaluated over one period According to Eilers and Peeters (1988), the specific growth rate of the microalgae culture, $\mu$, is proportional to the flux of transitions from the activated to the resting photosynthetic state, $\gamma x_{\textrm{A}}(t)$ , see (4), for the evaluation of specific growth rate over one period, and (3) for the value of the parameter $\gamma$. After *K* cycles, when $x_{\textrm{A}}(t)$ has reached its quasi-steady periodic regime, the cycle-averaged growth rate is

$$\begin{aligned} \mu \;=\;\frac{\kappa \gamma }{h}\int _{Kh}^{(K+1)h} x_{\textrm{A}}(t)\,\textrm{d}t, \end{aligned}$$

where $\kappa =3.647\times 10^{-3}$ for *Porphyridium* sp. Wu and Merchuk (2001). Following (Papáček et al. 2010), we evaluate a flashing schedule by its productivity, defined as the ratio between the time-averaged value of $x_A$ under a square-wave light profile and the steady-state abundance under continuous illumination at the same mean irradiance:

11 $$\begin{aligned} P \;=\; \frac{\displaystyle \frac{\mu }{\kappa \gamma }}{\,x_{\textrm{A}}^{\textrm{ss}}\!\bigl (u_{\textrm{av}}\bigr )}. \end{aligned}$$

With this convention, $P = 1$ corresponds to the ideal benchmark of continuous light delivering the same average photon flux, so $P < 1$ indicates a loss of performance due to flashing, whereas $P > 1$ would indicate a supra-optimal gain.

Kok’s flashing-light experiments of the 1950s (Kok 1953, 1956) reproduced rapid alternations and revealed that, once the flashes are sufficiently fast, the specific growth rate under on/off illumination approaches that under a continuous irradiance equal to the time average of the flashes. Subsequent studies – most notably those of Terry (1992) and Nedbal et al. (1996) – confirmed this intermittency principle, and a rigorous proof followed in Papáček et al. (2007): casting the PSF model as a single-input bilinear system, the authors showed that any two light signals with the same mean irradiance yield almost identical productivity, provided the period is short enough. Vigorous mixing in a PBR therefore guarantees productivity levels that are mathematically close to those of continuous illumination.^4^

Recent experiments, see e.g., Chiarini and Quadrio (2021); Inostroza et al. (2023), indicate that the hydrodynamically induced light–dark switching frequency, $f=1/h$, is not constant but random. To capture this variability, in Subsect. 2.3 we first derive the periodic solution of the deterministic model, and in Sect. 3 we then reformulate the reduced PSF system as a RDE with the period *h* treated as a random variable.

### Recursive solution for the reduced equation and periodicity

Because we are approximating the perceived light as a square wave, there is a recursive manner in which the proportion of active cells can be calculated. We consider the reduced equation (9) with input $u(t)$ given by (10), setting $u_a = 0$, which corresponds to the PSF being in complete darkness during the first portion of every period *h*.

For each $i\in \mathbb {N}$, let $\chi _{I_1^{(i)}}(t)$ and $\chi _{I_2^{(i)}}(t)$ denote the indicator function

$$ \chi _{I}(t) = {\left\{ \begin{array}{ll} 1, & \text {if } t \in I, \\ 0, & \text {otherwise.} \end{array}\right. } $$

of the intervals $I_1$ and $I_2$ (defined in (10)), which determine the domain of *u*(*t*).

Then, taking into account that $u_a=0$, the ODE governing the dynamics of $x(t)$ can be written compactly as

$$\begin{aligned} \dot{x}(t) = \left[ A x(t)\right] \chi _{I_1^{(i)}}(t) + \left[ B x(t) + C\right] \chi _{I_2^{(i)}}(t),\quad i=1,2,\ldots \end{aligned}$$

being

12 $$\begin{aligned} A&= - q_4 q_2<0, \end{aligned}$$

13 $$\begin{aligned} B&= - q_4 (u_b + q_2)<0, \end{aligned}$$

14 $$\begin{aligned} C&= q_4 u_b \frac{u_{\text {av}} + q_2}{q_2 u_{\text {av}}^2 + u_{\text {av}} + q_2}>0 . \end{aligned}$$

The explicit solution *x*(*t*) is given by

$$\begin{aligned} x(t) =\;&x_u^i e^{A (t - (i - 1)h)}\, \chi _{I_1^{(i)}}(t) \\&+ \left\{ \left( x_l^i e^{A r h} + \frac{C}{B} \right) e^{B (t - (i - 1)h - r h)} - \frac{C}{B} \right\} \ \chi _{I_2^{(i)}}(t), \nonumber \end{aligned}$$

where

$$ x_u^i := x((i - 1)h), \quad x_l^i := x((i - 1)h + r h),\quad i=1,2,\ldots $$

are the values of *x*(*t*) at the left-end and at the switching point of the *i*-th interval, respectively. The system satisfies the following transitions in the *i*-th interval

15 $$\begin{aligned} x_l^i&= x_u^i e^{A r h}, \end{aligned}$$

16 $$\begin{aligned} x_u^{i+1}&= x_l^i e^{B (1 - r)h} - \frac{C}{B} \left( 1 - e^{B (1 - r)h} \right) . \end{aligned}$$

Substituting equation (15) into (16) yields the recurrence relation

17 $$\begin{aligned} x_u^{i+1} = x_u^i e^{A r h + B (1 - r) h} - \frac{C}{B} \left( 1 - e^{B (1 - r) h} \right) . \end{aligned}$$

As previously shown, the model exhibits long-term periodic behavior regardless of the initial condition selected. Therefore, to ensure the system starts in a periodic regime, we choose the initial condition $x_0^p$ such that $x_0^p := x_u^{i+1} = x_u^i$ in equation (17). Solving for $x_0^p$ gives

18 $$\begin{aligned} x_0^p = x_0^p(h) = \frac{C \left( 1 - e^{B (1 - r) h} \right) }{-B \left( 1 - e^{A r h + B (1 - r) h} \right) }>0. \end{aligned}$$

Thus, the periodic solution can be written as:

19 $$\begin{aligned} \tilde{x}(t) =x_0^p e^{A (t - (i - 1)h)}\, \chi _{I_1^{(i)}}(t) + \left\{ \left( x_0^p e^{A r h} + \frac{C}{B} \right) e^{B (t - (i - 1)h - r h)} - \frac{C}{B}\right\} \chi _{I_2^{(i)}}(t). \end{aligned}$$

Let us define

20 $$\begin{aligned} \underline{x}(h)&:=e^{Arh}\frac{C \left( 1 - e^{B (1 - r) h} \right) }{-B \left( 1 - e^{A r h + B (1 - r) h} \right) }, \end{aligned}$$

21 $$\begin{aligned} \overline{x}(h)&:=\frac{C \left( 1 - e^{B (1 - r) h} \right) }{-B \left( 1 - e^{A r h + B (1 - r) h} \right) } . \end{aligned}$$

Observe that $\underline{x}(h)=e^{Arh}\overline{x}(h)$, with $A<0$, $r\in (0,1)$, so $\underline{x}(h)<\overline{x}(h)$ for $h>0$. Moreover, it is easy to see that at every time instant $t\ge 0$, the periodic solution $\tilde{x}(t)$ is bounded between two functions $\underline{x}(h)$ and $\overline{x}(h)$ explicitly given in (20) and (21), respectively,

22 $$\begin{aligned} \underline{x}(h)\le \tilde{x}(t)\le \overline{x}(h),\quad \forall t\ge 0. \end{aligned}$$

Indeed, from equation (19), one can see that it is monotonically decreasing in $I_1^{(i)}$ since $A<0$ and $x_0^p>0$ and it is monotonically increasing in $I_2^{(i)}$, since $x_0^pe^{Arh}+\frac{C}{B}>0$. When the system has reached its periodic regime, i.e., $x_u^{i+1} = x_u^i = x_0^p$, then the maximum value within each cycle occurs at the beginning of the cycle, at $t = (i-1)h$, and is given by $\underline{x}(h) = x_0^p e^{A r h}$ The minimum value occurs at the switching point $t = (i-1)h + rh$, and is given by $\overline{x}(h) = x_0^p$. Since $\tilde{x}(t)$ decreases over the subinterval $I_1^{(i)}$ until the switching point and increases over the subinterval $I_2^{(i)}$, its value at any point within the cycle remains within the bounds defined by (20) and (21), thus validating the inequality (22).

Notice that (22) gives a uniform envelope around the solution over each cycle. The bounds depend only on the period *h*, not on the specific point *t* in the cycle. So, no matter where the system is in time, the solution cannot escape the range defined by $\underline{x}(h)$ and $\overline{x}(h)$. This is a global-in-time stability statement, showing that the solution remains trapped inside an *h*-dependent band.

#### Remark 1

As demonstrated in (Celikovsky et al., 2010, Proposition 2.1), the PSF model (5) is biologically meaningful: the state variables remain nonnegative, and the sum of all state variables does not exceed one, i.e., $x_{\textrm{A}}(t) + x_{\textrm{B}}(t) \le 1$.

#### Remark 2

Let $\rho := A r + B(1-r), \, \tau := B(1-r),$ where $A<0$ and $B<0$ are defined in (12) and (13), respectively. Furthermore, recall the upper and lower envelopes $\overline{x}(h),\, \underline{x}(h)$ from (20)–(21). From these expressions, we can rewrite the envelopes as

$$ \overline{x}(h) = \frac{C(1 - \textrm{e}^{\tau h})}{-B(1 - \textrm{e}^{\rho h})}, \quad \underline{x}(h) = \textrm{e}^{A r h} \overline{x}(h). $$

It follows directly from L’Hôpital’s rule that

$$ \lim _{h \rightarrow 0} \underline{x}(h) = \lim _{h \rightarrow 0} \overline{x}(h) = -\frac{C \tau }{B \rho } = \frac{u_{\text {av}}}{q_2 \left( u_{\text {av}}^2 + u_{\text {av}}/q_2 + 1 \right) }. $$

Furthermore, if we consider $q_5 \ll 1$ (which is met in practice, since it is around $10^{-4}$ in our case study), then it is easy to see that

$$ -\frac{C \tau }{B \rho } \approx x_A^{\textrm{ss}}(u_{\text {av}}) = \frac{u_{\text {av}}}{q_2(1 + q_5)\left( u_{\text {av}}^2 + u_{\text {av}}/q_2 + 1 \right) }, $$

where $x_A^{\textrm{ss}}(\cdot )$ is the steady-state value defined in (7). This is expected: as the switching period *h* becomes vanishingly small, the input oscillates so rapidly between 0 and $u_b$ that, from the perspective of the system dynamics, it effectively experiences a constant input at the average value. Hence, the periodic solution converges to the same steady state that would be reached under continuous illumination at $u_{\text {av}}$. It connects the time-periodic regime to the classical autonomous system, offering a bridge between switching systems and their averaged behavior.

The following result characterizes the sensitivity of the solution to the switching frequency. It shows that higher frequencies suppress variation, lower frequencies amplify it. In other words, as the switching period *h* increases, the solution’s oscillations become larger: the upper bound rises, the lower bound drops. This means the system has more time in each mode (light or dark) to diverge from the average behavior, leading to wider fluctuations.

#### Lemma 1

Let $h\in (0,T^{*})$, with $T^*<\infty$. Then, the envelope bounds, $\overline{x}(h)$ and $\underline{x}(h)$ defined in (21) and (20), respectively, satisfy

$$ \overline{x}'(h)>0 \quad \text {and}\quad \underline{x}'(h)<0 \quad \text {for every }h\in (0,T^{*}), $$

that is, $\overline{x}$ is strictly increasing on $(0,T^{*})$ and $\underline{x}$ is strictly decreasing as functions of *h*.

#### Proof

Let us first define $\rho$ and $\tau$ as in Remark 2. Note that $\rho<\tau <0$ since $A<0$ and $r>0$. Then, $\overline{x}(h)$ and $\underline{x}(h)$ defined in (21) and (20), respectively, can be expressed as

$$ \overline{x}(h)=\frac{C \left( 1 - e^{\tau h} \right) }{-B \left( 1 - e^{\rho h} \right) }, \quad \underline{x}(h)=e^{Arh} \overline{x}(h). $$

We first justify the upper bound. Differentiating $\overline{x}$ once with respect to *h* gives

$$ \overline{x}'(h)=\frac{C}{-B}\, \frac{\rho e^{\rho h}-\tau e^{\tau h} +(\tau -\rho )e^{(\rho +\tau )h}}{(1-e^{\rho h})^{2}} =\frac{C}{-B}\; \frac{\phi (h)\,e^{(\rho +\tau )h}}{(1-e^{\rho h})^{2}}, $$

where $\displaystyle \phi (h):=\rho e^{-\tau h}-\tau e^{-\rho h}+(\tau -\rho ).$ Because $\phi (0)=0$ and $\phi '(h)=\rho \tau \bigl (e^{-\rho h}-e^{-\tau h}\bigr )>0$, we have $\phi (h)>0$ for all $h>0$. Since also $-C/B>0$, it follows that $\overline{x}'(h)>0$.

We now deduce the lower bound. Writing $\underline{x}(h)=e^{(\rho -\tau )h}\overline{x}(h)$ and differentiating,

$$ \underline{x}'(h) =e^{(\rho -\tau )h}\!\left[ \overline{x}'(h) +(\rho -\tau )\overline{x}(h)\right] =\frac{C}{-B}\,e^{(\rho -\tau )h}\, \frac{g(h)}{(1-e^{\rho h})^{2}}, $$

with $g(h):=(\rho -\tau )+\tau e^{\rho h}-\rho e^{\tau h}.$ Now $g(0)=0$ and $g'(h)=\rho \tau \bigl (e^{\rho h}-e^{\tau h}\bigr )<0$, so $g(h)<0$ for all $h>0$. Again using $-C/B>0$, we obtain $\underline{x}'(h)<0$.

Hence $\overline{x}(h)$ is strictly increasing and $\underline{x}(h)$ strictly decreasing for all $h\in (0,T^{*})$.

$$\square $$

The following result establishes that the pointwise bound given in (22) holds for all $t\in [0,h]$ and all $h\in ( 0,T^{*}]$. So, it can be considered as a reformulation of inequality (22) emphasizing that it holds uniformly across time and over a whole family of periods up to $T^{*}]$. It also shows that the envelope bounds are not just valid for a fixed period but they form a family of valid enclosures across a whole range of switching frequencies.

#### Corollary 1

Let $T^* > 0$, and suppose the switching period $h \in (0, T^*)$. Then, for all $t \ge 0$, the periodic solution $x(t)$ given in (19) satisfies the uniform bounds

$$ \underline{x}(T^*)< x(t) < \overline{x}(T^*), $$

where

$$ \overline{x}(T^*) = \frac{C \left( 1 - e^{\tau T^*} \right) }{-B \left( 1 - e^{\rho T^*} \right) }, \quad \underline{x}(T^*) = e^{(\rho - \tau ) T^*} \overline{x}(T^*), $$

with $\rho = A r + B(1-r)$, $\tau = B(1-r)$, satisfying $\rho< \tau < 0$, and *A*, *B* and *C* defined in (12)–(14).

**Fig. 4:**
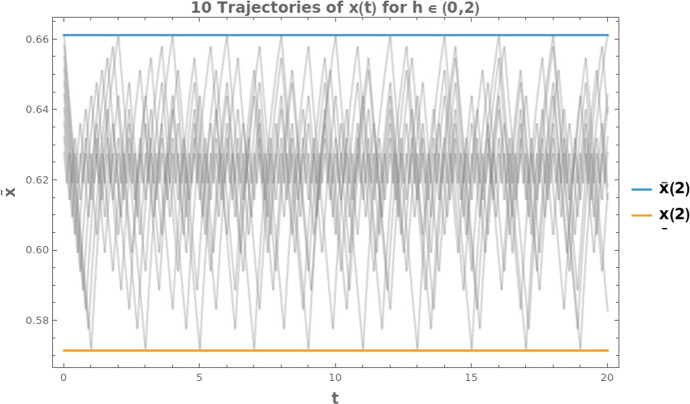
Ten trajectories of $\tilde{x}(t)$ are shown for $h \in (0,\, T^* = 2)$ over the time interval $t \in [0, 20]$ s. The blue line denotes the upper bound $\overline{x}(T^* = 2)$, while the orange line indicates the lower bound $\underline{x}(T^* = 2)$. The bounds are as shown in Corollary 1

Figure 4 presents a numerical example illustrating the uniform bounds (blue and orange lines). We simulate ten periodic trajectories of $\tilde{x}(t)$ (shown in gray) for $h \in (0, 2)\,\textrm{s}$ and $t \in [0, 20]\,\textrm{s}$. As *h* increases, the amplitude of $\tilde{x}$ also grows, with the largest envelopes occurring at $h = 2$. Most trajectories concentrate around approximately 0.62, and all remain within the upper and lower limits $\underline{x}(2)$ and $\overline{x}(2)$ indicated in the figure. This provides a visualization of both the boundedness of the trajectories and their accumulation around a mean value that is very close to $x_A^{ss}(1)$ (as defined in (7), with parameters for the microalga *Porphyridium* sp., $x_A^{ss}(1) \approx 0.62$), highlighting the average tendency of the random model and its finite variance.

#### Lemma 2

The periodic function $\tilde{x}(t)$, defined in (19), is Hölder continuous with exponent $p=1$ (that is, Lipschitz continuous) on $\mathbb {R}^+$.

The proof of this Lemma has been omitted since it follows immediately from the fact that $|\tilde{x}'(t)|$ is bounded. Indeed, on each subinterval $I_1^{(i)}$ and $I_2^{(i)}$, $\tilde{x}(t)$ is smooth, so Lipschitz; moreover, $\tilde{x}(t)$ has no jumps at subinterval endpoints, so is globally continuous, and $|\tilde{x}'(t)|$ is bounded, then is globally Lipschitz.

The following classical result, which will be key later on, can be concluded from this lemma and the periodicity of $\tilde{x}(t)$, more precisely $\tilde{x}(t)\in C^{0,1}_{\text {per}}([0,h])$ , i.e., (the space of periodic Lipschitz functions on [0, *h*]). The reader can refer to Theorem 8.12 in Teschl (n.d.).

#### Corollary 2

Let $S_n(\tilde{x})$ denote the $n$-th partial sum of the Fourier series of a the function $\tilde{x}$. If $\tilde{x} \in C^{0,1}_{\textrm{per}}([0,h])$ is Lipschitz continuous with period $H$, then its Fourier series converges uniformly. In particular,

$$ \Vert S_n(\tilde{x}) - \tilde{x} \Vert _{\infty } \;\longrightarrow \; 0 \qquad \text {as } n \rightarrow \infty . $$

#### Remark 3

As a consequence, classical results from Fourier analysis ensure that the Fourier coefficients of $\tilde{x}(t)$ decay at least as $\mathcal {O}(1/n^2)$ as $n \rightarrow \infty$. This decay will play a key role in the next section.

## Random model

At the end of Sect. 2.2, we have pointed out the intrinsic random nature of the parameter *h* (the light/dark cycle period), due to environmental factors. So, it makes more realistic to study the model from a stochastic standpoint. This motivates that in this section we move from the deterministic setting to a random one. To do so, let $(\Omega ,\mathcal F_{\Omega },\mathbb P)$ be a complete probability space and let $H:\Omega \longrightarrow (0,T^{*})$ be a real-valued absolutely continuous random variable, with probability density function (PDF) $f_{H}$, and $H\in L^2 (\Omega )$. For each fixed period $h\in (0,T^{*})$ the reduced model admits a deterministic periodic solution, which we denote by $x(t;h), \text { for } t \ge 0$ and whose explicit formula has been obtained in (19) (note that we drop the $\tilde{\cdot }$ notation and make the dependence on the value of *h* more obvious, i.e., $\tilde{x}(t):=x(t;h)$). We now define the *solution stochastic process* $\{X(t)\}_{t\ge 0}$ by

23 $$\begin{aligned} X(t;\omega )\;:=\;x\left( t;H(\omega )\right) , \quad t\ge 0,\;\omega \in \Omega . \end{aligned}$$

Thus all randomness in the real-valued stochastic process $X$ is entirely induced by the random period $H$ making $X(t;\omega )$ a *parametric* stochastic process. Noting that for each realization of *H*, $H(\omega )$, the stochastic process’s trajectory $t\mapsto x(t;H(\omega ))$ is completely deterministic. We denote by $x(t; h)$ the deterministic solution corresponding to a fixed real-valued parameter $h$. Throughout the paper, $X(t)$ refers to the general stochastic process, which will be used for the computation of statistical quantities such as expectation and variance. When we wish to emphasize a particular realization, we write $X(t; \omega )$, highlighting the dependence on the outcome $\omega \in \Omega$. Moreover, for a specific realization of the random variable $H$, we denote the associated deterministic solution as $x(t; H(\omega ))$, in order to emphasize its dependence on $H$.

In the next subsections, we address the computation of the main long-term characteristics of the randomized model, including its mean and variance.

### Expectation of the solution stochastic process

In Sect. 2.3, it was shown that, for every deterministic period $h\in (0,T^{*})$, the solution, $x(t;h)$, of the reduced model will eventually be $h$-periodic. If the initial state is chosen as $x_0^{p}(h)$ as defined in (18), the solution is *h*-periodic for all $t\ge 0$.

As previously indicated, in the random setting the period becomes the random variable $H:\Omega \rightarrow (0,T^{*})$. Choosing the random initial condition $x_0^{p}\bigl (H(\omega )\bigr )$ gives, for each $\omega \in \Omega$, $X(t;\omega )=x\left( t;H(\omega )\right)$, $t\ge 0$, so the path $t\mapsto X(t;\omega )$ is $H(\omega )$-periodic. To compute the expectation, we will assume that the deterministic mapping $h\mapsto x(t;h)$ is measurable and integrable in *h*, for fixed *t*.

By Corollary 2, the Fourier series of the deterministic solution $x(t;h)$ converges uniformly. Hence, for a fixed $h$, we can write

$$ x(t;h)=a_{0}(h)+\sum _{n=1}^{\infty } \Bigl [ a_{n}(h)\cos \bigl (2\pi n\tfrac{t}{h}\bigr )+ b_{n}(h)\sin \bigl (2\pi n\tfrac{t}{h}\bigr ) \Bigr ], $$

where

$$\begin{aligned} a_{0}(h)&=\frac{1}{h}\int _{0}^{h}x(s;h)\,\textrm{d}s,\\ a_{n}(h)&=\frac{2}{h}\int _{0}^{h}x(s;h)\cos \bigl (2\pi n\tfrac{s}{h}\bigr )\,\textrm{d}s,\\ b_{n}(h)&=\frac{2}{h}\int _{0}^{h}x(s;h)\sin \bigl (2\pi n\tfrac{s}{h}\bigr )\,\textrm{d}s. \end{aligned}$$

A closed-form expression for the coefficient $a_0$, that will play a key role later and corresponds to the time average of *x*(*t*; *h*) over a single period of length *h*, can be calculated using (19):

$$\begin{aligned} a_0(h) =&\, x_0^p(h) \left[ \frac{1}{B h} \left( -e^{A h r} + e^{A h r + B(h - h r)} \right) + \frac{1}{A h} \left( e^{A h r} - 1 \right) \right] \nonumber \\&+ \frac{C}{B^2 h} \left( e^{B(h - h r)} - 1 \right) - \frac{C}{B} (1 - r), \end{aligned}$$

where the constants $A$, $B$ and *C* are defined in (12), (13), and (14), respectively, and $x_0^p$ is defined in (18). Replacing $h$ by the random period $H(\omega )$ turns the Fourier coefficients into random variables $a_{n}(H(\omega ))$, $n=0,1,\ldots$, and $b_{n}(H(\omega ))$, $n=1,2,\ldots$, and yields, for a given $\omega \in \Omega$, the path-wise Fourier representation of the solution stochastic process

24 $$\begin{aligned} X(t;\omega )=a_{0}(H(\omega ))+ \sum _{n=1}^{\infty } \Bigl [ a_{n}(H(\omega ))\cos \bigl (2\pi n\tfrac{t}{H(\omega )}\bigr )+ b_{n}(H(\omega ))\sin \bigl (2\pi n\tfrac{t}{H(\omega )}\bigr ) \Bigr ]. \end{aligned}$$

From the definition of the expected value and the fact that the randomness in *X*(*t*) is inherited from *H*, we have that for each $t \ge 0$,

$$\begin{aligned} \mathbb {E}[X(t)] = \int _0^{T^*}x(t;h)f_H(h)\,\textrm{d}h. \end{aligned}$$

Considering the path-wise Fourier convergence, the expected value of *X*(*t*) for a given $t\ge 0$ can be written as

25 $$\begin{aligned} \mathbb {E}[X(t)] = \int _0^{T^*} \left( a_0(h) + \sum _{n=1}^\infty a_n(h) \cos \left( 2\pi n \frac{t}{h} \right) + b_n(h) \sin \left( 2\pi n \frac{t}{h} \right) \right) \, f_H(h) \, \textrm{d}h. \end{aligned}$$

In the following result, we will study of the long-term behavior of this expected value.

#### Theorem 1

For the stochastic process *X*(*t*) defined in (24), one gets

$$ \lim _{t\rightarrow \infty }\mathbb E[X(t)] \;=\; \int _{0}^{T^{*}} a_{0}(h)\,f_{H}(h)\,\textrm{d}h \;=\; \mathbb E\bigl [a_{0}(H)\bigr ]. $$

#### Proof

For $$n\ge 1$$ and $$h\in (0,T^{*})$$,

$$ |a_{n}(h)| \le \frac{2}{h}\int _{0}^{h}|x(s;h)|\,\textrm{d}s \le 2\,\overline{x}(T^{*}), $$

with $\overline{x}(T^{*})$ given in Corollary 1. Hence $a_{n}\in L^{\infty }(0,T^{*})$ and, since the PDF $f_H\in L^{1}(0,T^{*})$, then $a_{n}f_H,\;b_{n}f_H\in L^{1}(0,T^{*})$ by Hölder’s Inequality.

Let $\nu :=2\pi n/h$ (well-defined because $h>0$), hence $h=2\pi n/\nu$, and

$$ \int _{0}^{T^{*}}\!a_{n}(h)f_H(h)\cos \Bigl (2\pi n\tfrac{t}{h}\Bigr )\,\textrm{d}h =\int _{\frac{2\pi n}{T^{*}}}^{\infty }\! \alpha _{n}(\nu )\cos (\nu t)\,\textrm{d}\nu , $$

where $\alpha _{n}(\nu ):= a_{n}\!\bigl (\tfrac{2\pi n}{\nu }\bigr ) f_H\!\bigl (\tfrac{2\pi n}{\nu }\bigr ) \tfrac{2\pi n}{\nu ^{2}} \in L^{1}\!\bigl (\tfrac{2\pi n}{T^{*}},\infty \bigr ).$ The Riemann–Lebesgue lemma implies the integral tends to 0 as $t\rightarrow \infty$; the same holds for $b_{n}$ with $\sin (\cdot )$.

Because

$$ |a_{n}(h)|f_H(h),\,|b_{n}(h)|f_H(h) \le 2\overline{x}(T^{*})f_H(h)\in L^{1}(0,T^{*}), $$

the Dominated Convergence Theorem allows us to re-write the expected value as

$$\begin{aligned} \mathbb E[X(t)]&=\int _{0}^{T^{*}} \bigl [a_0(h)+ \sum _{n\ge 1}\!\bigl ( a_{n}(h)\cos (2\pi n\tfrac{t}{h})+ b_{n}(h)\sin (2\pi n\tfrac{t}{h}) \bigr )\bigr ]\,f_H(h)\,\textrm{d}h\\&=\mathbb E[a_{0}(H)]+R(t), \end{aligned}$$

where

$$ R(t):=\sum _{n\ge 1}\int _{0}^{T^{*}} (a_{n}(h)\cos (2\pi n\tfrac{t}{h})+b_{n}(h)\sin (2\pi n\tfrac{t}{h}))\,f_H(h)\,\textrm{d}h. $$

Moreover, each term in the series defining $R(t)$ tends to $0$ as $t\rightarrow \infty$ by the previous Riemann-Lebesgue argument, so it remains to show that we can interchange the limit and the infinite sum in $R(t)$, or equivalently, that the series $R(t)$ converges uniformly in $t$, or is absolutely summable uniformly in $t$, so we can conclude:

$$ \lim _{t \rightarrow \infty } R(t) = \sum _{n\ge 1} \lim _{t\rightarrow \infty } \int _{0}^{T^{*}} \bigl ( a_{n}(h)\cos (2\pi n\tfrac{t}{h}) + b_{n}(h)\sin (2\pi n\tfrac{t}{h}) \bigr ) f_H(h)\,\textrm{d}h = 0. $$

To this end, observe that:

$$ \left| \int _{0}^{T^{*}} a_{n}(h)\cos (2\pi n\tfrac{t}{h})\,f_H(h)\,\textrm{d}h \right| \le \int _{0}^{T^{*}} |a_{n}(h)|\,f_H(h)\,\textrm{d}h, $$

and similarly for $b_n$. Define:

$$ A_n := \int _{0}^{T^{*}} |a_{n}(h)|\,f_H(h)\,\textrm{d}h, \quad B_n := \int _{0}^{T^{*}} |b_{n}(h)|\,f_H(h)\,\textrm{d}h. $$

Since $|a_n(h)| \le 2\overline{x}(T^*)$, we get

$$A_n \le 2\overline{x}(T^*) \int _0^{T^*} f_H(h)\,\textrm{d}h = 2\overline{x}(T^*),$$

since $f_H$ is the PDF of *H*. And, similarly for $B_n$. Thus, using Remark 3, the series $\sum _{n\ge 1} |A_n + B_n|$ is convergent since is majorized by a constant multiple of the convergent series $\sum _{n\ge 1} 1/n^2$.

Therefore, the series $R(t)$ is uniformly absolutely convergent for all $t$, and each of its terms vanishes as $t\rightarrow \infty$. Hence, by the Weierstrass M-test, we conclude the proof. $\square$

#### Remark 4

*(Interpretation of Theorem* 1) The result of Theorem 1 is a manifestation of a classical averaging phenomenon. Although each realization of the process $X(t;\omega )$ is periodic, the randomness in the period *H* induces oscillatory terms of the form $\cos \!\left( 2\pi n t / H\right)$ and $\sin \!\left( 2\pi n t / H\right)$ whose frequencies depend on the random parameter *H*. As $t \rightarrow \infty$, these oscillatory terms become increasingly rapid functions of *h*, and their contributions cancel out when integrated against the probability density $f_H(h)$. This is a direct consequence of the Riemann–Lebesgue lemma (used in the proof itself) for highly oscillatory integrals. As a result, all nonzero Fourier modes vanish in expectation, and only the zero–frequency component $a_0(h)$, which corresponds to the time average of the deterministic periodic solution, contributes to the long–time expected value.

This type of phase–mixing or dephasing effect is well known in stochastic averaging, homogenization theory, and in systems with random frequencies.

### Variance of the solution stochastic process

For each $t\ge 0$, we have that the variance of the random variable *X*(*t*) is

$$ \operatorname {Var}[X(t)] = \mathbb {E}[( X(t) - \mathbb {E}[ X(t)])^2] = \mathbb {E}[ X(t)^2] - \mathbb {E}[ X(t)]^2, \, $$

where $\mathbb {E}[ X(t)^2] = \int _0^{T^*} {x}(t)^2 f_H(h)\,\textrm{d}h,$ and $\mathbb {E}[ X(t)]$ is given by (25). We will now proceed to bound this variance and study its long-time behaviour in the next two results.

The next result first establishes an envelope bound for $X(t;\omega )$ that holds almost surely (a.s.) provided that the random variable period $H\in (0,T^{*})$. Secondly, it provides a uniform bound for the variance of *X*(*t*) with respect to *t*.

#### Theorem 2

Let $H:\Omega \rightarrow (0,T^{*})$ be a continuous real-valued random variable and $X(t;\omega )$ the stochastic process defined by (23). Let $\rho = A r + B(1-r)$, $\tau = B(1-r)$ and *A*, *B* and *C* introduced in (12)–(14), define

$$ x_0^{p}(T^{*})\;=\; \frac{C\bigl (1-e^{\tau T^{*}}\bigr )}{-B\bigl (1-e^{\rho T^{*}}\bigr )}, $$

namely the value that (18) takes in the deterministic case $h= T^{*}$. Then, for every $t\ge 0$,

$$\begin{aligned} x_0^{p}(T^{*})\,e^{ArT^{*}} \;<\; {X}(t;\omega ) \;<\; x_0^{p}(T^{*}), \quad \forall \omega \in \Omega \text { a.s.} \end{aligned}$$

Moreover, the variance of *X*(*t*) satisfies the uniform bound

26 $$\begin{aligned} \operatorname {Var}\bigl [ {X}(t)\bigr ] \;\le \; \frac{C^{2}\bigl (1-e^{\tau T^{*}}\bigr )^{2}\bigl (1-e^{ArT^{*}}\bigr )^{2}}{4B^{2}\bigl (1-e^{\rho T^{*}}\bigr )^{2}}, \quad \forall \,t\ge 0. \end{aligned}$$

#### Proof

Corollary 1 states that the deterministic solution satisfies

$$ \underline{x}(T^{*})< x(t;h) < \overline{x}(T^{*}), \quad \forall \,h\in (0,T^{*}),\;\forall \,t\ge 0. $$

Replacing $h$ by the random variable $H(\omega )$ gives, for every $t\ge 0$ and $\omega \in \Omega$,

$$ \underline{x}(T^{*})< x(t;H(\omega )) < \overline{x}(T^{*}). $$

Now, we apply classical Popoviciu’s inequality on variances $\operatorname {Var}[Z]\le \frac{1}{4}(\sup Z-\inf Z)^2$ to $Z=X(t)\in (\underline{x}(T^{*}),\overline{x}(T^{*}))$ and obtain

$$ \operatorname {Var}[X(t)] \;\le \;\frac{1}{4}\! \bigl (\overline{x}(T^{*})- \underline{x}(T^{*})\bigr )^2 =\frac{1}{4}\bigl (1-e^{ArT^{*}}\bigr )^2 \bigl [x_{0}^{p}(T^{*})\bigr ]^2, $$

and substituting the closed form of $x_{0}^{p}(T^{*})$ gives exactly the right-hand side of (26). The estimate is uniform in $t$, so the proof is complete. $\square$

#### Remark 5

As $T^* \rightarrow +\infty$, the stochastic process *X*(*t*) tends to be bounded within the interval $[0,-C/B]$ a.s., and the variance tends to be upper bounded by ${{\,\textrm{Var}\,}}[ X(t)] \le C^2/(4 B^2)$. Moreover, when $T^* \rightarrow 0$, for every $\omega \in \Omega$, $X(t;\omega )$ tends to $\frac{-C (1-r)}{Ar + B(1-r)},$ which coincides with $\lim _{h \rightarrow 0^+} a_0(h)$, and ${{\,\textrm{Var}\,}}[ X(t)] \rightarrow 0$, so that the stochastic process *X*(*t*) becomes deterministic. The latter case ($h \rightarrow 0^+$) can be physically interpreted as the microalgae receiving continuous irradiance, and is taken as the reference case for comparing the growth of microalgae in photobioreactors. These two limit cases ($h\rightarrow 0$, i.e. $T^{*}\rightarrow 0$) and ($h\rightarrow \infty$, i.e. $T^{*}\rightarrow \infty$) correspond, respectively, to continuous light, deterministic behavior and zero variance, and to system saturates in the range $[0,-C/B]$, but random fluctuations persist with variance bounded by $C^2/(4B^2)$.

#### Theorem 3

For the continuous real-valued random value $H:\Omega \rightarrow (0,T^*)$ and the stochastic process *X*(*t*), it holds that

$$\lim _{t \rightarrow \infty }\operatorname {Var}[ {X}(t)] =\mathbb {E}[c_0(H)] - \mathbb {E}[a_0(H)]^2,$$

where

$$a_0 = \frac{1}{h} \int _0^h {x}(t) dt \quad \text {and} \quad c_0 = \frac{1}{h} \int _0^h {x}^2(t) dt.$$

#### Proof

At first, it should be noted that $$y(t) := {x}^2(t)$$ is Hölder continuous with exponent $$p=1$$ (Lipschitz continuous) on $$ \mathbb {R}^+ $$, since $$ {x}(t)>0$$ and $$\left| {x}'(t) \right| $$ are both bounded, so that

$$\left| y'(t)\right| = \left| \frac{\text {d}}{\text {d}t}\bigl ({x}(t)^2\bigr )\right| = 2 {x}(t) \left| {x}'(t) \right| $$

is also bounded. From here, it follows that *y*(*t*), which is also *h*-periodic and can be expressed as a Fourier series

$$\hat{y}(t) = c_0(h) + \sum _{n=1}^{\infty } c_n (h) \cos \left( 2\pi n \frac{t}{h} \right) + d_n (h) \sin \left( 2\pi n \frac{t}{h} \right) ,$$

and this series converges uniformly to *y*(*t*). By applying the same proof as Theorem 1, we can derive that

$$\lim _{t \rightarrow \infty }\mathbb {E}\left[ {X}^2(t)\right] = \int _0^{T^*} c_0(h)f_H(h) \, dh=\mathbb {E}[c_0(H)],$$

where

$$c_0(h) = \frac{1}{h} \int _0^h {x}^2(t) \,\textrm{d}t$$

does not depend on *t*. Therefore, it can be concluded that

$$ \lim _{t \rightarrow \infty }\operatorname {Var}[ X(t)] = \lim _{t \rightarrow \infty }\mathbb {E}\left[ X^2(t)\right] - \mathbb {E}\left[ X(t)\right] ^2 = \mathbb {E}[c_0(H)] - \mathbb {E}[a_0(H)]^2 $$

is constant. $$\square $$

#### Remark 6

Generally ${{\,\mathrm{\mathbb {E}}\,}}[a_{0}(H)^2]\ne {{\,\mathrm{\mathbb {E}}\,}}[c_{0}(H)]$, so ${{\,\textrm{Var}\,}}(a_{0}(H))$ does not equal the long-run variance $\lim _{t\rightarrow \infty }{{\,\textrm{Var}\,}}[X(t)]$. Let $\sigma ^2(h):= 1/h \int _0^h\bigl (x(t)-a_0(h)\bigr )^2\,\textrm{d}t$, Remark 2 tells us that the gap ${{\,\mathrm{\mathbb {E}}\,}}[\sigma ^{2}(H)]={{\,\mathrm{\mathbb {E}}\,}}[c_{0}(H)-a_{0}(H)^{2}]$ shrinks like as $T^*$ goes to 0. Thus, forcing $T^{*}$ very small makes $\lim _{t\rightarrow \infty }{{\,\textrm{Var}\,}}[X(t)]\approx {{\,\textrm{Var}\,}}(a_{0}(H))$, but at the cost of rendering $H$ almost deterministic, so the approximation is only meaningful in that limiting case.

### Numerical examples: influence of the flashing period and duty ratio on productivity

This subsection presents numerical experiments that quantify how the flashing period *H* and the light/dark duty ratio *r* affect the productivity of the PSF model.

For a deterministic period, productivity is defined in (11). When the period is treated as a random variable $H$, we extend that definition to

27 $$\begin{aligned} P(H)\;:=\;\frac{a_0(H)}{x_A^{\textrm{ss}}(u_{\textrm{av}})}, \end{aligned}$$

where $a_0(H)$ is the cycle-averaged concentration of the activated state obtained from the recursive solution in Sect. 2.3. Note that $a_0=\int _{Kh}^{(K+1)h}x(t)\text {d}t$ since we work with the initial condition that guarantees periodicity making $K=0$. The expected (mean) productivity is then

28 $$\begin{aligned} \mathbb {E}\!\bigl [P(H)\bigr ] \;=\; \frac{\mathbb {E}\!\bigl [a_0(H)\bigr ]}{x_A^{\textrm{ss}}(u_{\textrm{av}})} \;=\; \frac{\lim _{t \rightarrow \infty }\mathbb {E}[X(t)]}{x_A^{\textrm{ss}}(u_{\textrm{av}})}. \end{aligned}$$

It is important to note that through (28) we are able to relate the long-term mean behavior of the solution stochastic process *X*(*t*) and the average productivity rate *P*(*H*). In the remainder of this section we compute the mean, confidence intervals, and the PDF of *P*(*H*) for various probability distributions of *H* and duty ratios *r*, illustrating how randomness in the mixing frequency propagates to culture productivity. To model the flashing period, we first consider a uniform distribution, as it provides a non-informative prior when no strong assumptions can be made about which periods are more likely. It assigns equal probability across the admissible range, thereby avoiding the introduction of artificial bias. The Beta distribution serves as a natural generalization, since its flexible shape can be adapted to empirical evidence or expert knowledge. By tuning its two parameters, it can represent symmetric, skewed, or more concentrated behaviors, offering the flexibility required to capture more realistic variability when additional information is available.

We first let $H\sim \textrm{U}(0,T^{*})$ and vary the upper bound $T^{*}$ from $10^{-2}$ s to $\textrm{e}^{4}\!\approx \!54$ s, as indicated at the end of Subsect. 2.1 (the interval may be taken closed because $\mathbb {P}[H=0]=0$ for any continuous distribution).

**Fig. 5:**
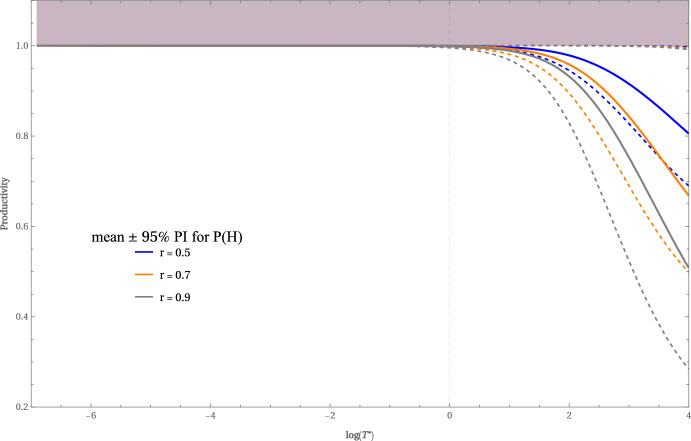
Mean and 95% CI of *P*(*H*) as defined in (27) versus $\log T^{*}$ for $H\sim \textrm{U}(0,T^{*}$) with $r=0.5$ and $r=0.9$.

In all numerical experiments we set the bright-phase intensity to

$$ u_{b}= \frac{1}{1-r}, \qquad u_{a}=0, $$

so that the time-averaged normalized irradiance is unity, $u_{\text {av}} = 1$. Figure 5 plots, for duty ratios $r = 0.5,\,0.7,$ and $0.9$, the mean productivity $P(H)$ (solid lines) together with its $95\,\%$ Monte-Carlo prediction interval (dashed lines) as a function of $\log T^{*}$, where the flash period is sampled as $H \sim U(0, T^{*})$. Each curve is based on $10^{6}$ independent simulations.

When flashes are extremely rapid, i.e. $\log T^{*}\ll 0$, all three mean curves and their prediction intervals collapse onto the ideal value of unity; virtually every simulation satisfies $0.99< P(H) < 1$. The dark gaps are too short for the photosystems to relax, so the cells behave as if they were under continuous light –precisely the behaviour first reported by Kok (1953, 1956)–. Once the upper limit of the flash period exceeds roughly $\log T^{*} = 1$, productivity begins to fall and does so more rapidly as the dark fraction $r$ increases. The schedule that spends half the time in light and half in darkness ($r = 0.5$) is the most robust: its mean productivity remains above $95\,\%$ of the optimum until $\log T^{*} \approx 1.5$ and is still near $80\,\%$ at $\log T^{*} \approx 4$. Introducing a moderate dark bias ($r = 0.7$) accelerates the decline –the mean stays close to unity only up to $\log T^{*} \approx 1$ and falls to about $70\,\%$ by $\log T^{*} \approx 4$–. When darkness dominates the cycle ($r = 0.9$) the loss is earliest and steepest: average productivity drops below $90\,\%$ shortly after $\log T^{*} = 0$, crosses the $70\,\%$ mark near $\log T^{*} = 2$ and slips under $50\,\%$ as $\log T^{*}$ approaches $4$.

The widening of the $95\,\%$ prediction intervals mirrors this trend. At $\log T^{*} = 4$ the intervals are $[0.690088,\,0.997584]$ for $r = 0.5$, $[0.499061,\,0.995126]$ for $r = 0.7$, and $[0.284728,\,0.991625]$ for $r = 0.9$. Each interval retains the nominal $95\,\%$ coverage $\bigl (0.95 + O(n^{-1/2})\bigr )$ with $n = 10^{6}$, and the associated standard error is $\sqrt{p(1-p)/n}$ with $p = 0.95$. Thus, as the flashing slows, cultures become increasingly vulnerable to dark-phase dominance: not only does the average productivity decline with larger $r$, but the uncertainty around that average grows as well.

Operationally, keeping $$T^{*}\lesssim \textrm{e}$$ (i.e. $$\log T^{*}\lesssim 1$$) secures, on average, at least 90 % of the theoretical yield even under a 9:1 dark–light ratio ($$r=0.9)$$. Once the duty cycle is fixed, flashing at high frequency is far more effective than adjustments in light fraction.

**Fig. 6:**
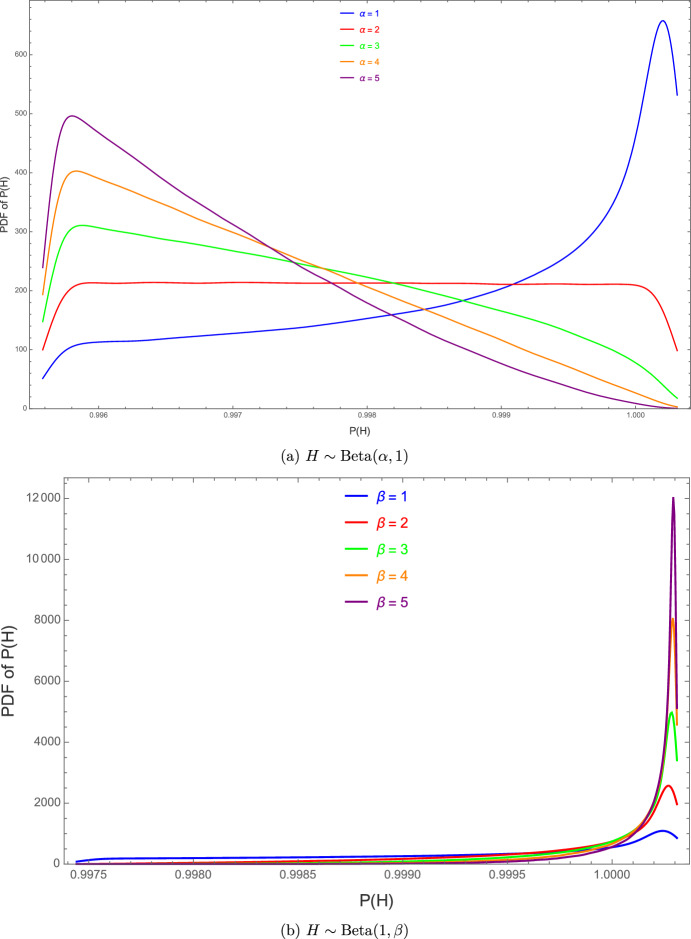
Probability density functions of the productivity *P*(*H*), defined in (27), when the flashing period *H* follows two complementary Beta distributions ($r=0.9$).

To isolate the impact of very short flashes with longer time spent in dark we fix the light–dark ratio at $r=0.9$ and draw the switching period from a Beta distribution on (0, 1). Figure 6 overlays $10^{6}$ kernel-smoothed realizations of the productivity random variable *P*(*H*) for two complementary Beta distributions. In the top panel the period follows $\textrm{Beta}(1,\beta )$; increasing $\beta$ pushes probability mass toward longer cycles, so the probability density of *P*(*H*) broadens and slides leftward away from the ideal value 1. In the bottom panel the period is sampled from $\textrm{Beta}(\alpha ,1)$; here a larger $\beta$ concentrates the mass near very short cycles, and the corresponding density of *P*(*H*) narrows dramatically while piling up at $P\approx 1$. Thus, when darkness dominates the duty cycle ($r=0.9$), shortening the period is far more effective at sustaining a high and predictable productivity than attempting to tune the light fraction alone.

## Conclusions

This work extends the three-state Photosynthetic Factory (PSF) model from a strictly deterministic setting to one in which the flashes of irradiance are perceived at randomly distributed intervals, an assumption that reflects what is actually measured in real industrial photobioreactors. By reformulating the reduced PSF system as a random differential equation, we derived closed-form expressions for the long-term mean and variance of the active cell state process and showed that the mean behaviour differs qualitatively from any single deterministic trajectory: while individual paths go to a quasi-steady wave pattern, the ensemble mean settles at a constant value.

Using Monte-Carlo simulations, we then quantified how randomness in the flashing period and changes in the light-to-dark duty ratio translate into productivity losses. The simulations confirm two practical guidelines. First, increasing the dark fraction inevitably lowers the mean productivity and enlarges its uncertainty. Second –and more importantly– keeping the maximum flashing period short is a far more effective way to preserve high, predictable yields than fine-tuning the duty ratio itself. In other words, vigorous mixing that speeds up light-zone cycling offers the most robust route to maximizing PSF productivity under real-world variability.

In practical terms, this points to design and operating strategies that minimise the longest residence times in dark or low-light zones, for example by appropriate choices of reactor geometry, mixing intensity and biomass concentration. The random differential equation (RDE)-based model can thus be used as a reliable tool to evaluate how sensitive a given photobioreactor configuration is to unavoidable variability in mixing and/or illumination.

Last, to the best of our knowledge, this is the first study that applies a RDE framework to describe microalgal growth in photobioreactors. While earlier studies have either characterised only the statistics of light/dark cycles, or have used a computationally demanding approach based on coupling a CFD model of the PBR with Lagrangian tracking of thousands of microalgal cells and PSF-type integrations along each irradiance history provided by the flow, our RDE-based formulation naturally couples the probability distribution of the light/dark period to an explicit dynamical growth model.

As a perspective for future work, the present RDE-based formulation could be extended toward a fully stochastic description in which the irradiance signal or the light/dark switching times evolve according to SDEs, allowing the incorporation of fast intra-cycle fluctuations. Such an extension would require more detailed experimental data on individual cell trajectories, which are currently not available to us, but would provide a qualitatively different and complementary description of photobioreactor dynamics.

## Data Availability

No data was used in the study. All parameter estimations come from Wu and Merchuk (2001).

## Footnotes

1 Cell density *N* and biomass concentration $c_x$ are related via the average cell dry mass $m_{\text {cell}}$, i.e., $c_x(t) \;=\; N(t)\, m_{\text {cell}},$ where $m_{\text {cell}}$ is of the order of $10^{-11}$–$10^{-12}$ g DW $\text {cell}^{-1}$ for many unicellular microalgae.

2 Because the primary photochemical reactions depend mainly on the number of photons absorbed in the photosynthetically active radiation (PAR) waveband, the term irradiance is frequently used, also reported as photosynthetic photon flux density (PPFD) in ${\upmu }$mol photons $\text {m}^{-2}$ $\text {s}^{-1}$, which corresponds to the number of photons in the PAR range per unit area and time.

3 Although system (1) is stiff for the parameter values in (3), it can be efficiently integrated with standard stiff ODE solvers. Our interest in reduced or averaged formulations is therefore not due to fundamental numerical limitations, but to the need to perform large ensembles of simulations over a wide range of light regimes.

4 At first sight, one might ask why we focus on light/dark sequences instead of considering a continuous illumination at a reduced intensity that provides the same time-averaged photon flux. In real PBRs, however, strong self-shading, technological and geometric effects create dark and low-light zones, so that cells traveling through the system perceive the light field as intrinsically intermittent, even under a continuous external source. Moreover, operating at a uniformly optimal continuous irradiance would require relatively dilute suspensions, whereas volumetric productivity increases with biomass concentration. A more realistic and industrially relevant strategy is to use a higher incident irradiance, e.g., PBR with Fresnel lenses as in Fig. 1 (left), and to work with dense cultures such that the *average* irradiance experienced by the cells is near the optimal value. Provided that the resulting light/dark cycles have a sufficiently high frequency, many microalgae strains can maintain near-optimal growth under such conditions.
